# Antrochoanal Polyp in Anterior Nasal Cavity: A Case Report

**DOI:** 10.7759/cureus.19823

**Published:** 2021-11-22

**Authors:** Yazeed Alghonaim, Faisal Bin Talib, Rana Alramyan, Buthaina J Yahya, Mohammad Alkraidees, Abdullah AlKarni

**Affiliations:** 1 Otolaryngology, King Saud Bin Abdulaziz University for Health Sciences College of Medicine, Riyadh, SAU

**Keywords:** ent, surgical case reports, otorhinolaryngology, antrochoanal polyp, polyp

## Abstract

We report a case of antrochoanal polyp, which has unusual presentation according to the location of the polyp in a 15-year-female patient. The patient came complaining of nasal obstruction, headache, and postnasal drip for a two-week period. The antrochoanal polyp measured 2.5 x 2 cm in the left maxillary sinus and extended to the anterior part of the nasal cavity. CT imaging demonstrated a total opacified left maxillary sinus, maxillary ostium with widening of the left maxillary ostium by polypoid mucosal thickening suggesting an antrochoanal polyp obstructing the left anterior nasal cavity. This case is reported as there are not many articles in world literature describing an antrochoanal polyp presented in the anterior nasal cavity.

## Introduction

Antrochoanal polyp (ACP) is a solitary benign growth, and it usually originates from the lining of the maxillary antrum. It typically grows by extension from the antrum through its ostium into the middle meatus, after that it extends into the posterior choana and it might reach the nasopharynx [1]. ACPs usually occur solitary and are seen mainly in young adults (less than 40 years) [2]. ACPs represent only 5% of sinonasal polyps, and are more common in males [3].

## Case presentation

The patient, a 15-year-old female, complained of nasal obstruction, headache, and post-nasal discharge for two weeks. The patient had no significant past medical history, nor a history of allergy. Physical examination of the patient revealed a huge nasal polyp occupying the left nasal cavity pushing the nasal septum to the right. In addition, there was a thick yellowish discharge on physical examination.

**Figure 1:**
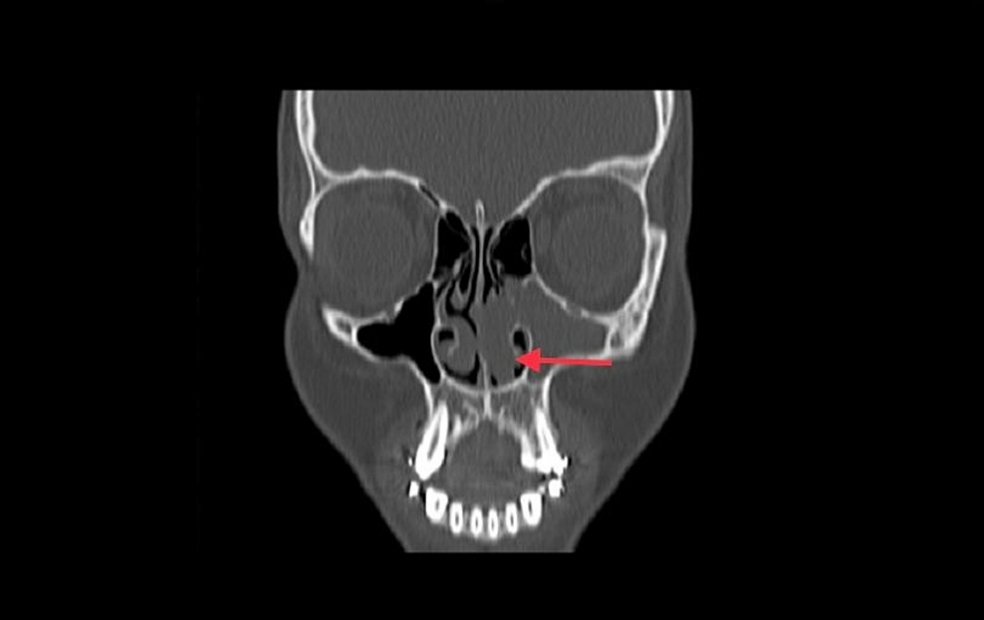
Coronal view of left antrochoanal polyp arising from the maxillary sinus and extending to the anterior nasal cavity.

**Figure 2:**
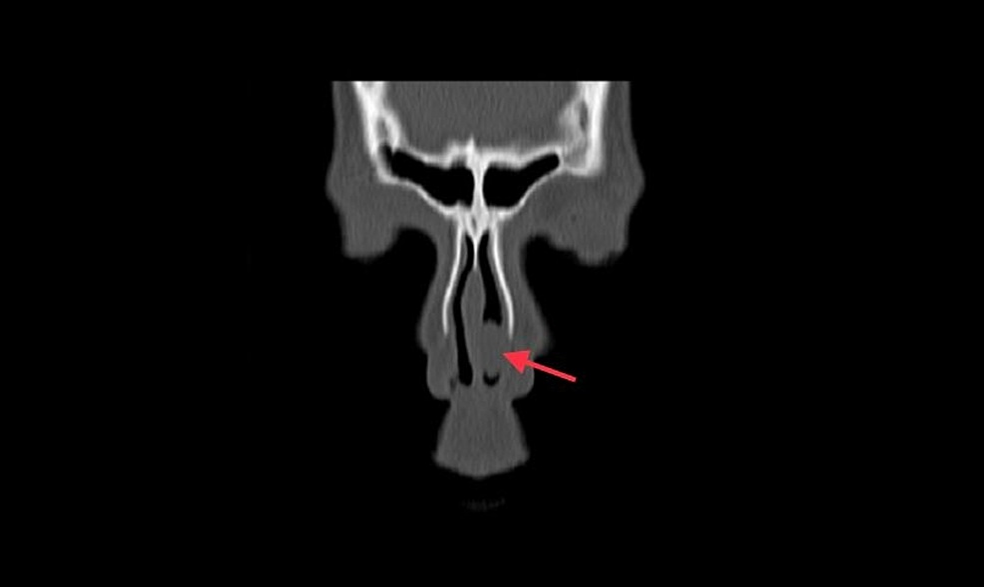
Coronal view of left antrochoanal polyp.

**Figure 3:**
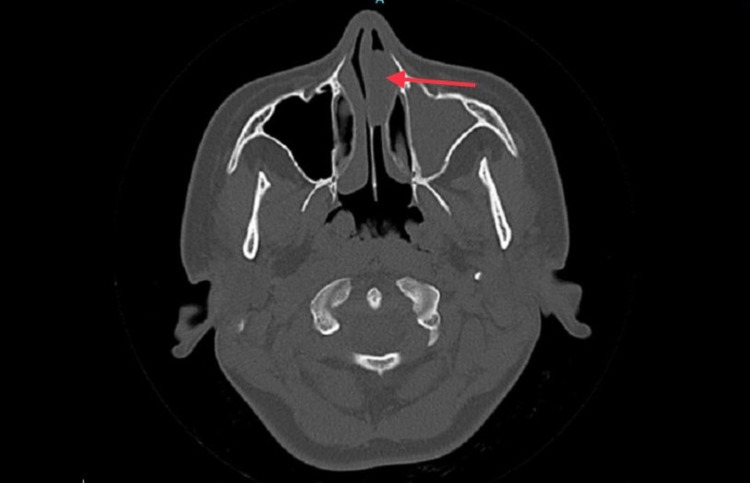
Axial view of left antrochoanal polyp obstructing anterior nasal cavity.

Non-enhanced computerized tomography (CT scan) of the paranasal sinuses was performed. The sphenoid, ethmoidal, and frontal sinuses were clear and unremarkable. The left maxillary sinus showed a total opacification with polypoidal mucosal thickening and mild remodeling seen at the bony outline. Furthermore, there was opacification and widening of the left maxillary ostium with mucosal thickening surrounding the uncinate process, infundibulum, and hiatus semilunaris of the left side extending to the lateral aspect of the mid to anterior left nasal cavity. On the other hand, the right maxillary sinus, right maxillary ostium, right uncinate process, infundibulum, and hiatus semilunaris were unremarkable. The nasal cavity showed mild circumferential smooth thin mucosal thickening related to rhinitis with a maximal thickness of 1 mm. Additionally, a large (2.5 x 2 cm) polypoidal mucosal thickening was noted obstructing the anterior to mid part of the mid to inferior left nasal cavity communicating with the mucosal thickening of the left maxillary antrum. Also, the nasal septum was diffusely deviated to the right side, and there was no involvement of the soft tissue neither the nasopharynx. Moreover, the left middle meatus was partially opacified by mucosal thickening, and the middle concha was partially paradoxical at the left side. The right inferior concha was moderately to significantly hypertrophied, while the left inferior concha was displaced and indented by the polypoidal mucosal thickening at the nasal cavity (Figures 1-3).

## Discussion

ACPs are benign soft tissue lesions that arise from the maxillary sinus. They pass through sinus ostium and grow into the choana and might extend to the nasopharynx. It was first described by Professor Gustav Killian, in 1906, giving them a specificity among polyposis [2,4]. They usually affect people less than 40 years old and are usually solitary and unilateral [5].^ ^The main two symptoms are nasal obstruction and nasal discharge, yet in severe cases the symptoms can also include epistaxis, dyspnea, dysphagia, and weight loss [6,7].

In histopathology the macroscopic features mainly consist of a cystic part filling the maxillary sinus and a solid part coming out through the maxillary ostium into the middle meatus and subsequently to choana. Microscopically, the polyp shows a central cavity surrounded by homogeneous edematous stroma bearing a few cells [8].

ACPs are mainly diagnosed by CT imaging due to their precise bony details of the paranasal sinuses. The typical finding in CT scans consists of a defined mass with mucin density that arises from maxillary sinus, widening of the maxillary ostium and extending into the nasopharynx, and smooth enlargement of the sinus without bony destruction [9]. In our case, all of the imaging features are present except for the unusual location of the polyp anteriorly. Treatment of ACPs is complete surgical removal of the polyp through an operation called functional endoscopic sinus surgery (FESS).

We presented our case report due to the rare presentation of the ACP, which usually presents from the antrum through its ostium into the middle meatus, and after that it extends into the posterior choana. In contrast, in our case, it originated from the maxillary sinus through the ostium and went anteriorly to the anterior part of the nasal cavity against gravity without any obvious reason.

## Conclusions

ACPs usually occur in people less than 40 years old.

Commonly, ACPs originate from the maxillary sinus and extend toward the posterior choana. However, in our case, the ACP extended to the anterior nasal cavity against gravity without any obvious reason.

FESS is the appropriate treatment of ACP in contrast to more invasive open procedures that are reserved for severe cases that cannot be managed endoscopically.
